# Implementing the Indigenous Diabetic Foot Project in the lower gulf of Australia

**DOI:** 10.1186/1757-1146-4-S1-P46

**Published:** 2011-05-20

**Authors:** Caroline Radowski, Catherine Willett, Courtney Thomas, Rahni Wisely

**Affiliations:** 1 NWQPHC, Mount Isa, Queensland, 4825, Australia; 2 Queensland Health, Mount Isa, Queensland, 4825, Australia

## 

North and West Queensland Primary Health Care (NWQPHC) is a division of general practice which provides allied health services to rural and remote north-west Queensland. The area covered by this organisation spans 770 000km2 and has a resident population of approximately 114 000 people. Podiatrists working within this region provide an innovative primary health care model based upon the Ottawa Charter principals. Podiatrists work in a multidisciplinary team and travel to each community every four weeks. Diabetes effects large proportion of the Indigenous population and is responsible for approximately 3000 foot amputations a year. In acknowledgment of this a primary health care initiative was implemented in Normanton located in the Lower Gulf of Australia. This program was run in collaboration with a Queensland Health Podiatrist and was aimed at training Indigenous Health Workers in screening diabetic feet. By implementing the diabetic project Indigenous Health Workers could pre-screen diabetic feet and recognise the risk a client has of developing an ulcer. They could also recognise foot problems that would require medical attention or the treatment of a Podiatrist. By training staff in this field it was hoped that eventually there would a community member always available to address diabetic foot concerns while the Podiatrist is not in the community and it would recognise high risk feet to the Podiatrist attention before any complication could develop. The work shop was run over two days and there were seven Indigenous Health Workers that participated in the course. The Indigenous Health Workers consisted of NWQPHC, Queensland Health and Home and Community Care Staff. There were six females and one male that attended the course and all participates completed the course successfully. The program was based around SARRAH’s course material and taught the participants to be able to: (i) care for feet, (ii) check people’s feet, (iii) finding pulses on the foot, (iv) using a monofilament, (v) understand the difference between high risk and low risk feet, (vi) teach clients the basics of self care, (vii) complete a DART form (assessment tool), (viii) understand the referral process for a high risk foot. All participants initially learnt the course work and information about diabetic feet and later were able to practice on each other. The following day several known diabetic clients attended the program for the participants to assess them as if they were a new client. The participants each felt pulses, used a monofilament noted any areas of concern on the feet and filled out a DART form classifying the patient as high risk or low risk. All participants were able to complete each screening technique on each client and are now currently using the DART assessment tool as a standard form for all Diabetic Clients. It is hoped that more Indigenous Health Workers are trained in this course to continue to help the fight against diabetic foot complications. In the future NWQPHC in collaboration with Queensland Health would like Indigenous Health Workers Trained in each community to use the DART assessment form. This would empower the community to take control over the diabetic problem and hopefully decrease the rates of foot amputations through early high risk detection. If this program was successful in the long term the potential of training Indigenous Health Workers as foot assistance has also been explored.

